# Accuracy of the Kirchhoff-Approximation and Kirchhoff-Ray-Mode Fish Swimbladder Acoustic Scattering Models

**DOI:** 10.1371/journal.pone.0064055

**Published:** 2013-05-14

**Authors:** Gavin J. Macaulay, Héctor Peña, Sascha M. M. Fässler, Geir Pedersen, Egil Ona

**Affiliations:** 1 Observation Methodology, Institute of Marine Research, Bergen, Norway; 2 Wageningen Institute for Marine Resources and Ecosystem Studies (IMARES), IJmuiden, The Netherlands; 3 CMR Instrumentation, Christian Michelsen Research AS, Bergen, Norway; Pacific Northwest National Laboratory, United States of America

## Abstract

The acoustic backscatter from pressure release prolate spheroids and a three-dimensional representation of a fish swimbladder (Chilean jack mackerel, *Trachurus symmetricus murphyi*) was calculated using four target strength models (Kirchhoff-approximation, Kirchhoff-ray-mode, finite element solution of the Helmholtz equation, and prolate-spheroid-modal-series). Smoothly varying errors were found in the Kirchhoff-approximation and Kirchhoff-ray-mode model results when compared to the other models, and provide objective criteria for constraining the use of the KA and KRM models. A generic correction technique is also proposed for the prolate spheroid estimates and tentatively tested on a jack mackerel swimbladder, resulting in improvements to the target strength estimates from the Kirchhoff-approximation and Kirchhoff-ray-mode models.

## Introduction

Estimates of the acoustic reflectivity of fish (target strength, TS) are an important input to acoustic surveys of fish populations [1]. TS measurements of fish at the same time and location as the survey are preferable, but can be difficult and time-consuming to achieve. Acoustic scattering models are a complementary technique for estimating TS that can provide an enhanced understanding of variation with variables such as species, size, shape, and acoustic frequency. Many species of fish possess a gas-filled swimbladder, used for regulating buoyancy and sound reception and generation [2]. This gas presents a high acoustic contrast, which causes the swimbladder reflection to dominate the backscatter from fish at moderate frequencies [3].

Many acoustic scattering models have been developed or adapted to simulate the scattering from gas-filled swimbladders, which can be conveniently assumed to reflect as a soft (pressure-release) surface. These include the *T*-matrix method [4], various formulations of scattering from straight and deformed cylinders (e.g., [5], [6], [7]), the use of the Kirchhoff approximation in various forms [8], [9], the prolate-spheroid-modal-series model [10], the Fourier mode matching method [11], and the solution of the Helmholtz equation using the boundary element method [12] and the finite element method [13]. It is important that models give results that are representative of scattering from fish, therefore much work has been done to validate models by comparison with *in situ* and *ex situ* measurements. Overall, there is reasonable agreement (e.g., [14], [15], [16], [17]), but differences between modelled and measured TS of several decibel (dB) are common at broadside and off-broadside angles (e.g., [15], [18], [19], [20], [21], [22]).

Two commonly used scattering models are the Kirchhoff-approximation model (KA) [9] and the Kirchhoff-ray-mode model (KRM) [8]. Both make use of the Kirchhoff approximation [23] and operate on a three-dimensional representation of the swimbladder. The KA model approximates the swimbladder by a closed surface of planar facets, which can represent any swimbladder shape given sufficiently small facets, while the KRM model uses a set of stacked and potentially offset cylinders and approximates the swimbladder by an object with a piecewise circular cross section. The KRM uses an empirical correction to the Kirchhoff approximation to improve the accuracy at low frequencies and a low mode solution for very low frequencies [8], [24]. Due to the Kirchhoff approximation, both models become less accurate at non-normal reflection angles [25] and a maximum off-broadside angle of 25 to 45 degrees has been used in some swimbladder studies [8], [9].

The investigation and selection of appropriate criteria for the use of scattering models is typically left to the model users and the models can be unknowingly used in situations where their performance is poor. This will lead to inaccurate TS estimates and associated errors in fish biomass estimates (e.g., a 3 dB underestimate in TS can result in a biomass estimate twice the actual). There are few published comparisons of the KA and KRM models to an exact solution at commonly used acoustic frequencies and swimbladder shapes and sizes for pressure release surfaces [12]. Rather, comparison of TS estimates from several swimbladder models to each other, or to *ex situ* or *in situ* measurements, are more common. The latter has the attraction of comparing the models to the “correct” result, but does not necessarily demonstrate that the model itself is functioning correctly, nor whether it is being used within its physical and numerical limitations. Examples of comparisons include McClatchie *et al.* [26], who applied three models to three species of fish and found differences in tilt-averaged TS between 0 and about 5 dB. Jech *et al.* [19] applied three scattering models to *ex situ* measurements from one species of fish and found broad agreement, but with significant differences in some cases. Sawada *et al.* [22] compared two models with *ex situ* measurements from two species of fish and also found significant differences between the models and measurements.

This paper uses the KA and KRM models to estimate the TS of pressure release prolate spheroids (which approximate a fish swimbladder) and compares them to estimates from the analytically exact prolate-spheroid-modal-series model (PSMS) [10] at a range of aspect ratios and frequencies. The error in the KA and KRM TS estimates is calculated and can be used to retrospectively improve the output from KA and KRM models. The error estimates also provide clear constraints on the use of the KA and KRM models. A correction that can be applied to fish swimbladders rather than just prolate spheroids is also desirable. As a first approximation, the prolate spheroid-derived correction is applied to a Chilean jack mackerel (*Trachurus symmetricus murphyi*) swimbladder using the finite element (FE) solution of the Helmholtz equation as the reference solution with the performance of the FE model demonstrated via comparison to the PSMS model. The implementation of the four models (KA, KRM, PSMS, and FE) in computer code is validated by comparison to theoretical results for the KA and FE models, and to published model results for the PSMS and KRM models.

## Methods

The backscattered TS from prolate spheroids with semi-major axis *a* and semi-minor axis *b* was calculated using the KA, KRM, PSMS, and FE models at a range of incident acoustic wave angles. A length-normalised TS was calculated as 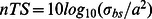 [dB], where 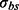 [m^2^] is the backscattered cross-section, and *a* [m] the semi-major axis [27].

Fifteen sets of prolate spheroids were used, where *ka* (*k* is the acoustic wavenumber, equivalent to 2*πf/c* where *f* is the acoustic frequency [Hz] and *c* the sound speed [m s^−1^]) ranged from 0.5 to 20 and *kb* varied from 0.25 to 10. These gave aspect ratios (*a/b*) ranging from 1.2 to 80, covering the swimbladder aspect ratios of many acoustically surveyed fish species (e.g., [10], [21], [28], [29]). The length-normalised TS was calculated for each prolate spheroid at incident angles from 0 to 50 degrees in 2 degree or finer steps, where 0 degrees was the broadside direction. A tilt-averaged length-normalised TS, <*nTS*> was then calculated for each prolate spheroid using a normal tilt distribution with mean of 0 degrees and standard deviation of 10 degrees, and subtracted from the corresponding PSMS <*nTS*> value to give an estimate of the error in each model. Where required by the model, the sound speed in the water surrounding the prolate spheroid was set to 1479.6 m s^−1^ and the density to 1027 kg m^−3^. The acoustic frequency was fixed at 38 kHz and the prolate spheroid sizes chosen to achieve the desired *ka* and *kb* values.

The KA method was implemented as per Foote & Francis [12] for a pressure release surface and used triangular facets with edge lengths that were always less than 1/16 of a wavelength. The KRM method was implemented as per Clay & Horne [8] and divided the prolate spheroid into cylinders that were 0.05 mm thick for *ka* ≥2.5 and 0.01 mm thick for *ka* <2.5. The transition between the Kirchhoff-ray approximation and low mode solution in the KRM model occurs at a mean *kb* of 0.2– all of the prolate spheroids used here were above that value and the low mode component was not utilised. The KRM method is typically used to simulate a gas-filled body [15], but to better match the pressure release surface of the other models the density and sound speed inside the prolate spheroid were set to zero, thereby giving a pressure release surface. The PSMS method was implemented as per Furusawa [10] for a pressure release surface and was used as the reference solution. The PSMS model is numerically challenging at higher *ka* due to the evaluation of spheroidal wave functions and the requirement for convergence of the summation of an infinite series with terms of oscillatory magnitude. Because of this, Furusawa [10] only presents results up to *ka*  = 12, but by using more recent algorithms [30] this study could calculate solutions up to *ka*  = 20. This limit is not relevant to the other models, but to provide full comparability the other models were also limited to *ka*  = 20.

The FE model solutions were calculated using the finite element method, as implemented in the COMSOL Multiphysics software package [31], which numerically solved the three-dimensional Helmholtz equation for scattering from a pressure release surface [13], [32], [33]. The scattering objects were surrounded by a spherical volume of water, itself surrounded by a spherical volume that absorbed the radiating acoustic energy using a perfectly matched layer one wavelength in thickness [34], [35]. Linear and quadratic Lagrangian finite elements were used with at least 10 nodes per wavelength to adequately resolve the acoustic waves. The far-field backscattered TS was calculated using the Helmholtz-Kirchhoff integral on the boundary between the spherical water volume and perfectly matched layer.

In addition to the prolate spheroids, the TS from a three-dimensional representation of a Chilean jack mackerel swimbladder (specimen 20 [21]) was estimated using the PSMS, KA, KRM, and FE methods at 38 kHz. The swimbladder model was constructed from magnetic resonance images of a fish and had a length of 67.9 mm, maximum height of 9.7 mm and maximum width of 10.3 mm. The PSMS model used a semi-major length equal to half the length of the swimbladder and a semi-minor width equal to half the mean of the maximum height and width. A tilt offset of –15 degrees (thereby moving the head down) was applied to the PSMS swimbladder results, corresponding to the average of measured swimbladder dorsal surface inclinations [21]. The KA and FE used a smoothed version of the swimbladder surface (Laplacian smoothing, [36]) with ≥24 facets or nodes per wavelength. The KRM divided the swimbladder into 133 circular slices.

The implementation of the models in computer code was validated by comparing model outputs to exact solutions or published results from the relevant model. The KA and FE implementations were validated by calculating the scattering from a 25 mm radius sphere with pressure release surface, immersed in a liquid with density of 1025 kg m^−3^ and sound speed 1470 m s^−1^, as per Foote and Francis [12]. The normalised backscattered TS was calculated at 1 to 200 kHz, corresponding to *ka* values of 0.1 to greater than 20. The KA model output was compared to the analytical solution of the Kirchhoff integral for the sphere [12] and the FE model output was compared to the exact series solution for the scattering from a pressure release sphere [37]. The PSMS implementation was validated by comparison to the results presented in Figure 3 of Furusawa [10] – a prolate spheroid with *b/a* aspect ratio of 0.15 and pressure release surface at 0.25≤ *ka* ≤12, evaluated at broadside and end-on backscatter angles. The KRM implementation was validated by comparison to Figure 11 of Reeder and Stanton [11], which presents the backscatter from a pressure release axisymmetric object at a range of angles and frequencies. The object shape was digitised and input to our KRM implementation.

## Results and Discussion

### Model validation

**Figure 1:**
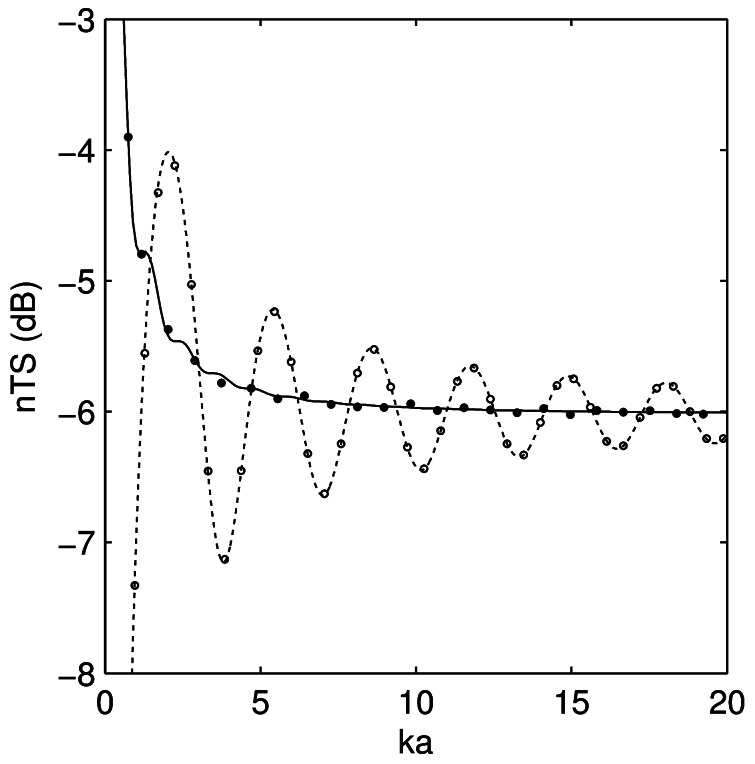
Comparison of the Kirchhoff-approximation and finite element models to exact solutions. Length-normalised target strength (*nTS*) of a 25 mm diameter pressure release sphere immersed in water as a function of *ka*, calculated using the Kirchhoff approximation model (open circles), Kirchhoff integral (dashed line), finite element model (filled circles) and series solution (solid line).

**Figure 2:**
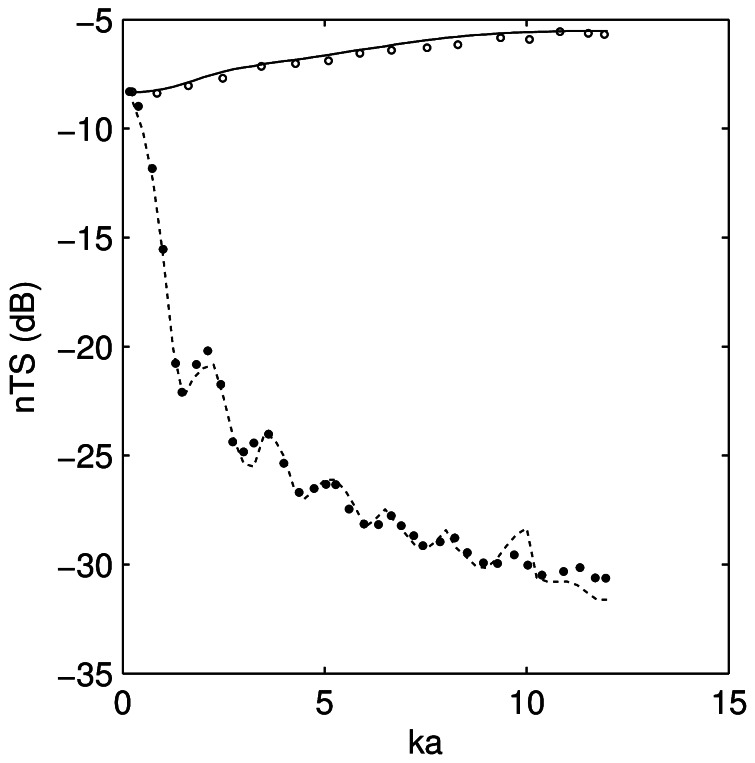
Comparison of prolate-spheroid-modal-series model to published results. Length-normalised target strength (*nTS*) of a pressure release prolate spheroid with aspect ratio (width/length) of 0.15 at broadside as a function of *ka* from the prolate-spheroid-modal-series model (solid line) and digitised from Figure 3 of Furusawa [10] (open circles). A similar comparison for end-on backscatter is also shown (dashed line from the prolate-spheroid-modal-series model and closed circles from Figure 3 of Furusawa [10]).

**Figure 3:**
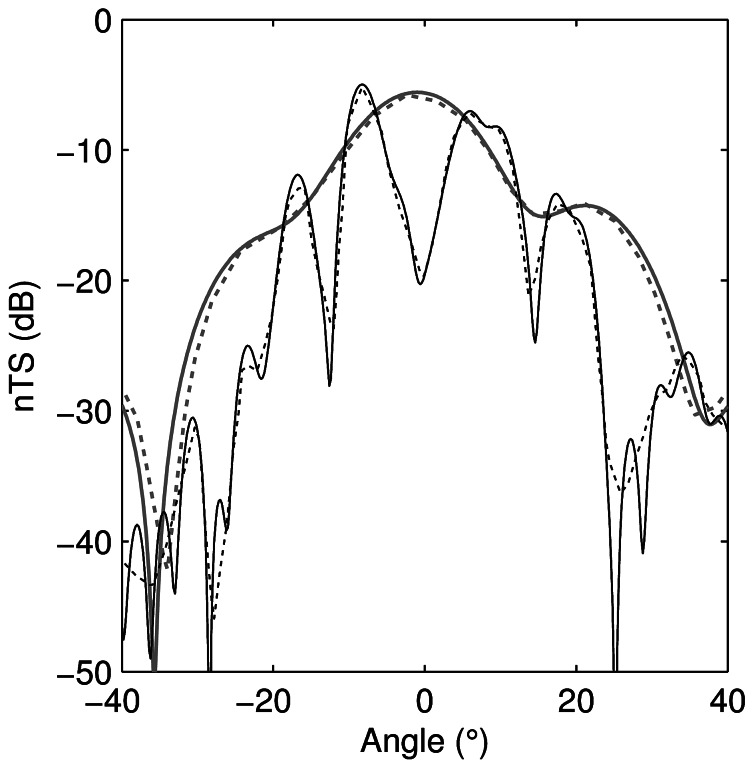
Comparison of Kirchhoff-ray-mode model to published results. Length-normalised target strength (*nTS*) of the axisymmetric shape given in Figure 11 of Reeder and Stanton [11] calculated by the Kirchhoff-ray-mode model (solid lines) and digitised from Figure 11 of Reeder and Stanton [11] (dotted lines) for *kb*  = 1 (grey) and *kb*  = 5 (black). The implementation of this model used a finer angle resolution than by Reeder and Stanton [11] and shows more precisely the null responses in the backscatter.

The KA results agreed to within 0.03 dB root mean square (RMS) of the analytical solution of the Kirchhoff integral for the sphere over the frequency range (Figure 1). The FE results agreed to within 0.04 dB RMS of the series solution for the sphere over the frequency range (Figure 1). Of particular concern with the FE model is the use of a sufficiently fine mesh to yield an accurate solution. This is typically demonstrated with a convergence test, where the solution is estimated several times with increasingly finer meshes until the result converges. For the sphere, this occurred with 9–10 elements per wavelength. The PSMS results agreed to within 0.2 and 0.5 dB RMS of Figure 3 in Furusawa [10] for broadside and end-on backscatter respectively (Figure 2).

A good correspondence was achieved between the KRM model and those presented in Figure 11 of Reeder and Stanton [11] (Figure 3), albeit with increasing differences at off-broadside angles, which are attributed to inaccuracies in the manual digitisation of the object shape. Agreement for the –20 to 20° angle range was within 0.3 dB and 1.3 dB RMS for *ka*  = 1 and *ka*  = 5 respectively. The angular resolution used in our KRM results was finer than that in Reeder and Stanton [11], giving improved resolution of the deep nulls. This lead to a larger RMS value for the *ka*  = 5 result, where several nulls occur within the –20 to 20 degree angle range.

### Model comparison

**Figure 4:**
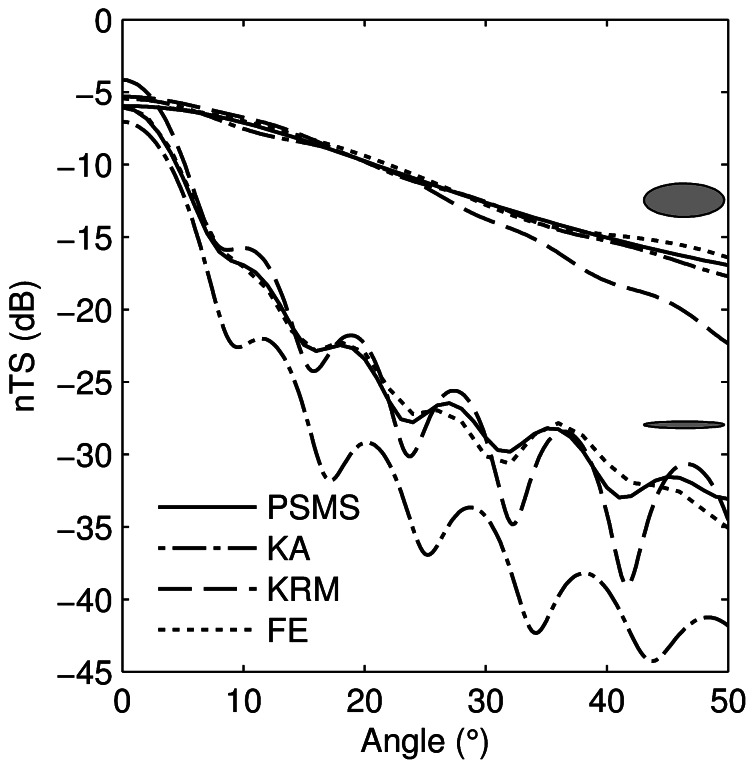
Comparison of model results for two prolate spheroids. Length-normalised target strength (*nTS*) of a prolate spheroid with *ka*  = 12 and *kb*  = 5 (upper curves) and *ka*  = 12 and *kb*  = 1 (lower curves) as a function of angle off broadside from the prolate-spheroid-modal-series (PSMS), Kirchhoff-approximation (KA), Kirchhoff-ray-mode (KRM), and finite element (FE) models, where *a* is the semi-major axis and *b* the semi-minor axis of the prolate spheroid. The shaded ellipses show the relative shapes of the prolate spheroids.

The general performance of the four models is illustrated with two examples. For a thick prolate spheroid (*ka*  = 12, *kb*  = 5, Figure 4, upper curves), all models gave similar TS until about 25 degrees off broadside, where the KRM starts to diverge, to eventually give a 5 dB TS underestimate at 50 degrees. At broadside of a thinner prolate spheroid (*ka*  = 12, *kb*  = 1, Figure 4, lower curves) the FE and PSMS models are very similar, while the KA and KRM models differ from the PSMS by less than 2 dB until about 8 degrees. The KRM tracks the PSMS well as angle increases except for the magnitude of the dips, where it is lower, while the KA increasingly diverges from the PSMS as angle increases. These characteristics were generally present for all of the simulated values of *ka* and *kb*. Provided that *ka* ≥2 and *kb* ≥2, the simulations indicated that all models agreed to within 2 dB for angles off broadside up to 30 degrees. At smaller *kb*, all models were accurate up to at least 30 degrees, except for the KA. At *kb*  = 1, the KA was accurate out to 5–10 degrees, but for smaller *kb*, errors of several dB occurred. The FE results tracked the PSMS results well at all angles and aspect ratios (Figure 4).

**Figure 5:**
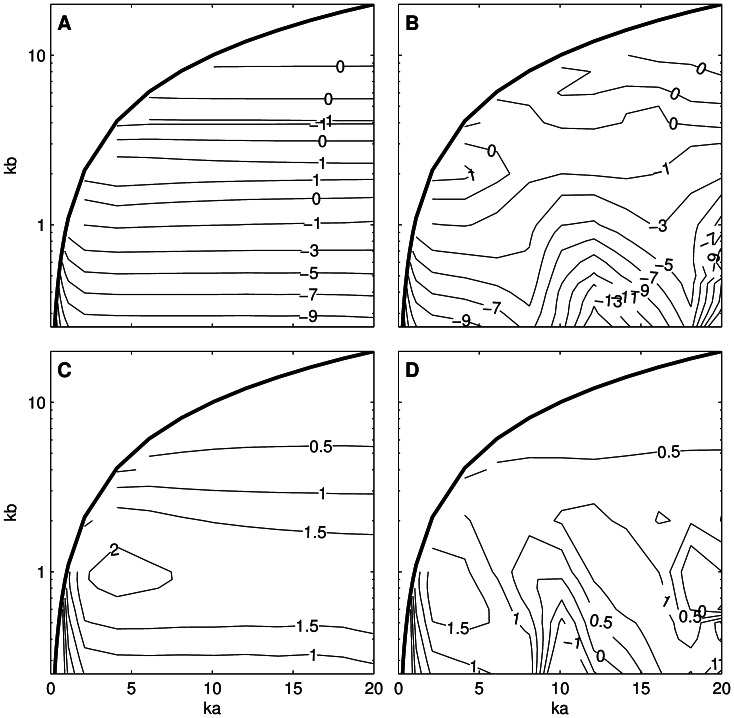
Target strength error from the Kirchhoff-approximation and Kirchhoff-ray-mode models at two tilt angles. Difference in length-normalised target strength for the Kirchhoff-approximation (KA) and Kirchhoff-ray-mode (KRM) and models compared to the prolate-spheroid-modal-series model as a function of *ka* and *kb* at a tilt angle of 0° (panel A: KA, panel C: KRM) and 10° (panel B: KA, panel D: KRM). Negative values indicate that the model result is less than the prolate-spheroid-modal-series model result. The thick solid line is drawn at *ka* = *kb*.

The difference between the KA and PSMS estimates of *nTS* at broadside is almost constant for a given *kb*, provided that *ka* >2 (Figure 5, panel A). Differences larger than 5 dB were found when *kb* ≤0.5. This is consistent with an analysis of the Kirchhoff approximation, which postulates that, to give a good result, the product of *k* and the minimum radius of curvature of the surface be much greater than one [25]. It is clear that for prolate spheroids this is the semi-minor axis (*kb*) rather than the semi-major axis (*ka*). However, “much greater than one” is conservative; values as low as one can yield reasonable broadside TS estimates for prolate spheroids with the KA method. The differences between the KA and PSMS at tilt angles away from broadside can be considerably larger (e.g., greater than 13 dB at 10° tilt) and show a periodic variation with increasing *ka* (Figure 5, panel B), due in part to nulls in the backscatter occurring at differing angles (Figure 4). The differences between the KRM and PSMS model results have broadly similar characteristics to the KA, but the difference was always less than 2.1 dB at broadside and at 10° (Figure 5, panels C and D, respectively).

**Figure 6:**
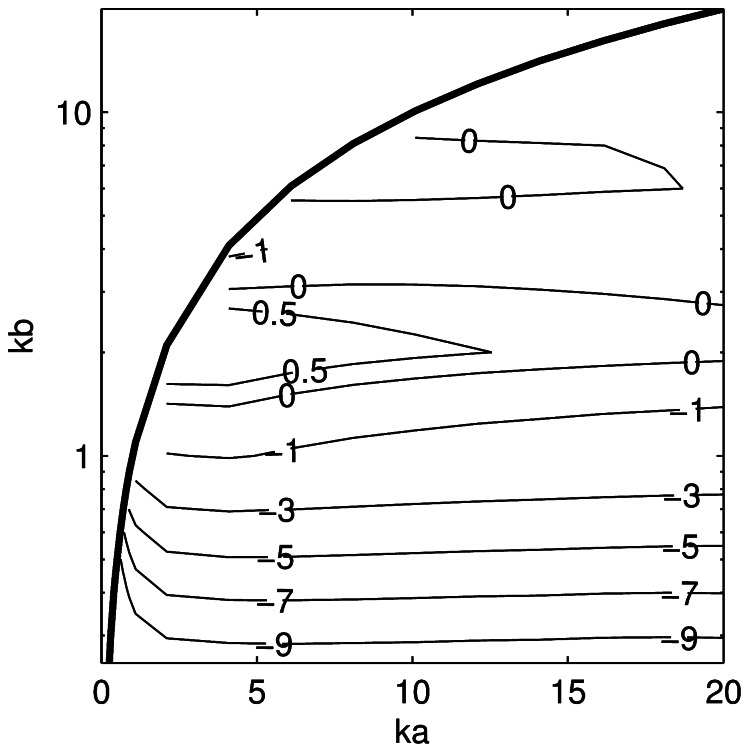
Target strength error for the Kirchhoff-approximation model. Difference in tilt-averaged (mean of 0° and standard deviation of 10°) length-normalised target strength between the Kirchhoff-approximation and prolate-spheroid-modal-series models as a function of *ka* and *kb* (negative values indicate that the Kirchhoff-approximation model result is less than the prolate-spheroid-modal-series model). The thick solid line is drawn at *ka* = *kb*.

**Figure 7:**
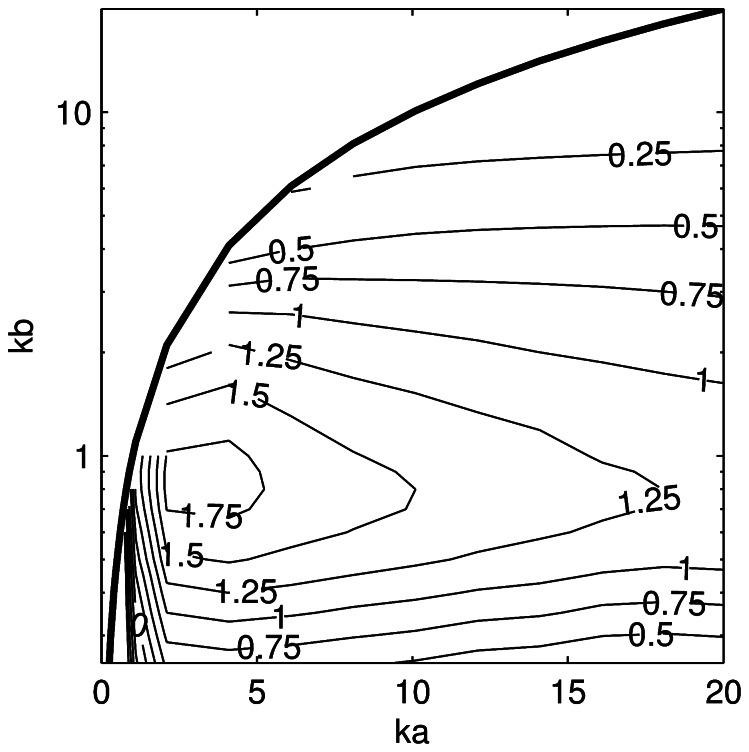
Target strength error for the Kirchhoff-ray-mode model. Difference in tilt-averaged (mean of 0 and standard deviation of 10°) length-normalised target strength between the Kirchhoff-ray-mode and prolate-spheroid-modal-series models as a function of *ka* and *kb* (positive values indicate that the Kirchhoff-ray-mode model result is greater than the prolate-spheroid-modal-series model). The thick solid line is drawn at *ka* = *kb*.

The difference between the KA and PSMS estimates of <*nTS*> as a function of *ka* and *kb* (Figure 6) is reasonably constant for a given *kb* when *ka* >2. Differences larger than 5 dB were found when *kb* ≤0.5. The differences between the KRM and PSMS estimates of <*nTS*> (Figure 7) have broadly similar characteristics to the KA, but the difference was always less than 2 dB when *ka* >1 and *kb* >0.25. The FE estimates of *nTS* and <*nTS*> (neither shown) were always within 0.7 dB of the PSMS value and improved slightly with lower *ka* and *kb*. Close agreement to the PSMS results depended to a large degree on using a sufficiently fine FE mesh.

The KRM derived estimates of <*nTS*> were almost always higher than the PSMS, while the KA estimates were almost always lower than the PSMS (Figures 6 and 7). There were only small variations in the differences when the tilt-angle distribution was changed and these results are appropriate for tilt angle distributions with means between 0 and 10 degrees and standard deviations between 0 and 20 degrees. They can therefore be used as a generic adjustment to tilt-averaged prolate spheroid TS estimates from the KA and KRM models. The underlying data (Table S1) can be interpolated to obtain an adjustment for arbitrary *ka* and *kb* within the region investigated here (0.5<*ka* ≤20, 0.25<*kb* ≤10, and *ka* >*kb*).

The Kirchhoff approximation is based on a ray-optics approach [38] and does not account for acoustic diffraction [25], a phenomenon whereby the acoustic wave bends around an object. Diffraction is more prevalent when the wavelength is of a size similar to, or larger than, the object. This corresponds to low *ka* or *kb* values for a prolate spheroid. It is postulated that the increasing error in the KA model at low *ka* and *kb* is due to the neglect of diffraction and that this lack is countered in the KRM model by the use of an empirical correction term, which was explicitly introduced to improve the solution at small object dimensions relative to the wavelength [8], [24]. The Kirchhoff approximation also gives poor results at large non-normal backscatter angles [25], although the effect is small on the tilt-averaged results presented here due to the restricted angle distribution used for the tilt averaging.

A method for retrospectively correcting fish swimbladder TS estimates from the KA and KRM models is desirable as many estimates have been published using these models. A preliminary technique is to assume that a fish swimbladder approximates a pressure release prolate spheroid [10] and to use the prolate spheroid corrections presented above. This is tested on the Chilean jack mackerel swimbladder, which at 38 kHz has *ka* and *kb* of 5.5 and 0.8, respectively. The error estimates (Figures 6 and 7) indicate that the tilt-averaged TS from the KA and KRM models should differ from a prolate spheroid of the same dimensions by –2.2 and 1.7 dB, respectively, and due to the low *kb* value the KA model is perhaps not appropriate for this swimbladder and frequency.

**Figure 8:**
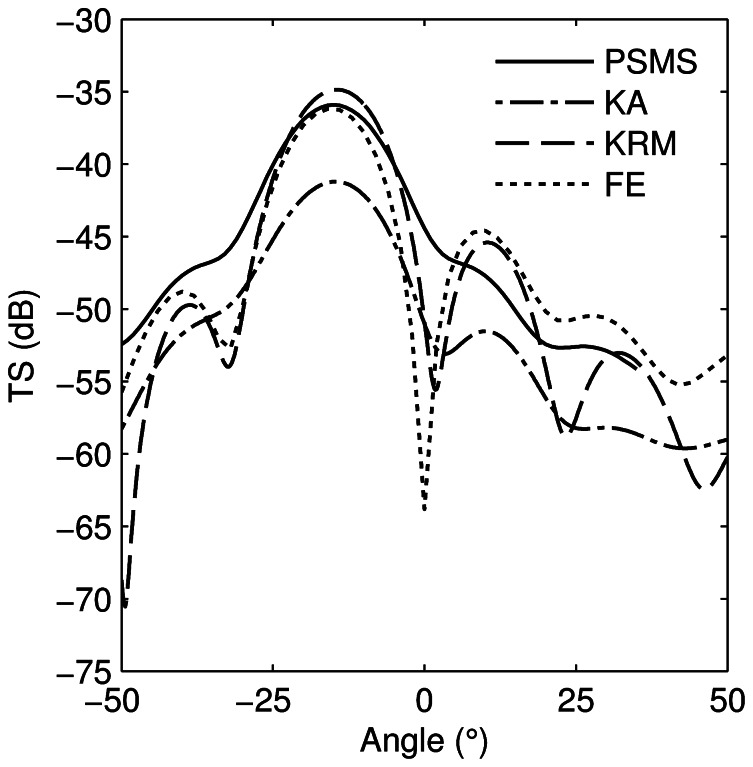
Chilean jack mackerel target strength. Prolate-spheroid-modal-series (PSMS), Kirchhoff-approximation (KA), Kirchhoff-ray-mode (KRM), and finite element (FE) model target strength estimates of the Chilean jack mackerel swimbladder at 38 kHz as a function of tilt angle. The prolate-spheroid-modal-series results have been offset by –15 degrees to match the average tilt of the dorsal surface of the swimbladder. Positive angles indicate a fish head up tilt.

All models gave broadly similar TS-tilt responses for the swimbladder (Figure 8) and agreed to within 2 dB at broadside except for the KA, which was about 5 dB lower. At angles away from maximum TS the correspondence with the reference model (FE in this case) decreased. The tilt-averaged target strengths (using a tilt distribution with mean of –15° and standard deviation of 10°) for the swimbladder were –38.2 dB (PSMS), –43.5 dB (KA), –37.6 dB (KRM), and –39.0 dB (FE). The KA underestimated the reference model TS by 4.5 dB and the KRM overestimated it by 1.4 dB. While the estimated correction for the KA was 2.3 dB less than the actual difference, it was in the correct direction and reinforces the earlier statement that the KA results may be inaccurate due to the low *kb*. The correction for the KRM was within 0.3 dB of the actual difference. Further testing of this correction technique is required on a range of swimbladders as well as the development of a metric to evaluate whether a given swimbladder can be treated as a prolate spheroid-like shape.

The FE model gave results similar to the PSMS in all prolate spheroid model runs, but has the significant disadvantage of requiring large computational resources for three-dimensional simulations. This is particularly so at higher frequencies because of the need to have a mesh density capable of resolving the acoustic wave. This hinders its use for fish TS modelling. The computational requirements for the KA, KRM, and PSMS models are minimal in comparison.

The results presented here are for a pressure release surface, as is commonly used with the KA, PSMS, and FE fish swimbladder models. The KRM model typically uses a gas-filled swimbladder with appropriate values for density and sound speed of the gas. However, for typical values (such as air at atmospheric pressure), there is little difference in the backscatter from a gas-filled swimbladder and a pressure release swimbladder, and for consistency the KRM results presented here are from a pressure release surface.

Each swimbladder TS model has constraints on its use, particularly the region of validity and expected accuracy; the choice of a model for any particular swimbladder should be made with a full awareness of these. The work presented here clarifies the region of validity of the KA and KRM models when applied to prolate spheroid pressure release surfaces and provides a method to improve the accuracy of the KA and KRM models with minimal effort. Improved accuracy will lead to more representative TS estimates and thus to improvements in acoustic estimates of fish population biomass.

## Supporting Information

Table S1 **Errors estimates for Kirchhoff-approximation and Kirchhoff-ray-mode scattering models for a range of prolate spheroids.** Errors estimates for the tilt-averaged [0, 10] prolate spheroid target strength (dB) calculated by the Kirchhoff-approximation (KA) and Kirchhoff-ray-mode (KRM) scattering models for a range of prolate spheroids with semi-major dimension *ka* and semi-minor dimension *kb*. Errors are obtained by comparison to the tilt-averaged TS calculated from the prolate spheroid modal series model. These data can be interpolated to yield error estimates for any *ka* and *kb* within the region investigated (0.5<*ka*≤20, 0.25<*kb*≤10, and *ka* >*kb*).
(DOC)
