# Supplementary material for: Accuracy of the Kirchhoff-Approximation and Kirchhoff-Ray-Mode Fish Swimbladder Acoustic Scattering Models
Source: PLoS One. 2013 May 14;8(5):e64055. doi: 10.1371/journal.pone.0064055 (PMC3653833; doi:10.1371/journal.pone.0064055)
Supplement: Table S1 — Errors estimates for Kirchhoff-approximation and Kirchhoff-ray-mode scattering models for a range of prolate spheroids. Errors estimates for the tilt-averaged [0, 10] prolate spheroid target strength (dB) calculated by the Kirchhoff-approximation (KA) and Kirchhoff-ray-mode (KRM) scattering models for a range of prolate spheroids with semi-major dimension ka and semi-minor dimension kb. Errors are obtained by comparison to the tilt-averaged TS calculated from the prolate spheroid modal series model. These data can be interpolated to yield error estimates for any ka and kb within the region investigated (0.5<ka≤20, 0.25<kb≤10, and ka >kb). (DOC) [file pone.0064055.s001.doc]

**Table S1. Errors estimates for Kirchhoff-approximation (KA) and Kirchhoff-ray-mode (KRM) scattering models for a range of prolate spheroids.**

| *ka* | *kb* | KA | KRM |
| --- | --- | --- | --- |
| 0.3 | 0.25 | -16.6 | -5.7 |
| 0.35 | 0.25 | -15.8 | -4.9 |
| 0.35 | 0.3 | -14.9 | -5.1 |
| 0.4 | 0.25 | -15.1 | -4.3 |
| 0.4 | 0.3 | -14.2 | -4.4 |
| 0.4 | 0.35 | -13.4 | -4.5 |
| 0.45 | 0.25 | -14.5 | -3.7 |
| 0.45 | 0.3 | -13.6 | -3.8 |
| 0.45 | 0.35 | -12.8 | -3.9 |
| 0.45 | 0.4 | -12.1 | -3.9 |
| 0.5 | 0.25 | -14.0 | -3.3 |
| 0.5 | 0.3 | -13.1 | -3.3 |
| 0.5 | 0.35 | -12.3 | -3.4 |
| 0.5 | 0.4 | -11.5 | -3.4 |
| 0.5 | 0.45 | -10.8 | -3.4 |
| 0.6 | 0.25 | -13.2 | -2.4 |
| 0.6 | 0.3 | -12.2 | -2.4 |
| 0.6 | 0.35 | -11.4 | -2.4 |
| 0.6 | 0.4 | -10.6 | -2.3 |
| 0.6 | 0.45 | -9.8 | -2.3 |
| 0.6 | 0.5 | -9.1 | -2.2 |
| 0.7 | 0.25 | -12.6 | -1.8 |
| 0.7 | 0.3 | -11.6 | -1.7 |
| 0.7 | 0.35 | -10.7 | -1.7 |
| 0.7 | 0.4 | -9.8 | -1.6 |
| 0.7 | 0.45 | -9.1 | -1.6 |
| 0.7 | 0.5 | -8.4 | -1.5 |
| 0.7 | 0.6 | -7.1 | -1.4 |
| 0.8 | 0.25 | -12.1 | -1.2 |
| 0.8 | 0.3 | -11.0 | -1.2 |
| 0.8 | 0.35 | -10.1 | -1.1 |
| 0.8 | 0.4 | -9.3 | -1.0 |
| 0.8 | 0.45 | -8.5 | -0.9 |
| 0.8 | 0.5 | -7.8 | -0.8 |
| 0.8 | 0.6 | -6.5 | -0.7 |
| 0.8 | 0.7 | -5.4 | -0.5 |
| 0.9 | 0.25 | -11.7 | -0.9 |
| 0.9 | 0.3 | -10.6 | -0.8 |
| 0.9 | 0.35 | -9.6 | -0.7 |
| 0.9 | 0.4 | -8.8 | -0.6 |
| 0.9 | 0.45 | -8.0 | -0.5 |
| 0.9 | 0.5 | -7.3 | -0.4 |
| 0.9 | 0.6 | -6.0 | -0.2 |
| 0.9 | 0.7 | -4.9 | 0.0 |
| 0.9 | 0.8 | -4.0 | 0.1 |
| 1.1 | 0.25 | -11.1 | -0.3 |
| 1.1 | 0.3 | -9.9 | -0.1 |
| 1.1 | 0.35 | -8.9 | 0.0 |
| 1.1 | 0.4 | -8.1 | 0.1 |
| 1.1 | 0.45 | -7.3 | 0.2 |
| 1.1 | 0.5 | -6.5 | 0.4 |
| 1.1 | 0.6 | -5.3 | 0.5 |
| 1.1 | 0.7 | -4.2 | 0.7 |
| 1.1 | 0.8 | -3.3 | 0.8 |
| 1.1 | 0.9 | -2.6 | 0.8 |
| 1.1 | 1 | -2.1 | 0.8 |
| 2.1 | 0.25 | -10.1 | 0.6 |
| 2.1 | 0.3 | -8.9 | 0.8 |
| 2.1 | 0.35 | -7.8 | 1.0 |
| 2.1 | 0.4 | -6.9 | 1.2 |
| 2.1 | 0.45 | -6.1 | 1.3 |
| 2.1 | 0.5 | -5.3 | 1.5 |
| 2.1 | 0.6 | -4.1 | 1.6 |
| 2.1 | 0.7 | -3.1 | 1.8 |
| 2.1 | 0.8 | -2.3 | 1.8 |
| 2.1 | 0.9 | -1.6 | 1.8 |
| 2.1 | 1 | -1.0 | 1.8 |
| 2.1 | 2 | 1.4 | 1.1 |
| 4.1 | 0.25 | -9.9 | 0.6 |
| 4.1 | 0.3 | -8.6 | 0.9 |
| 4.1 | 0.35 | -7.6 | 1.1 |
| 4.1 | 0.4 | -6.6 | 1.3 |
| 4.1 | 0.45 | -5.8 | 1.4 |
| 4.1 | 0.5 | -5.1 | 1.5 |
| 4.1 | 0.6 | -3.9 | 1.7 |
| 4.1 | 0.7 | -2.9 | 1.8 |
| 4.1 | 0.8 | -2.1 | 1.8 |
| 4.1 | 0.9 | -1.5 | 1.8 |
| 4.1 | 1 | -0.9 | 1.8 |
| 4.1 | 2 | 1.4 | 1.3 |
| 4.1 | 4 | -1.3 | 0.3 |
| 6.1 | 0.25 | -9.8 | 0.6 |
| 6.1 | 0.3 | -8.6 | 0.8 |
| 6.1 | 0.35 | -7.6 | 1.0 |
| 6.1 | 0.4 | -6.6 | 1.2 |
| 6.1 | 0.45 | -5.8 | 1.3 |
| 6.1 | 0.5 | -5.1 | 1.4 |
| 6.1 | 0.6 | -3.9 | 1.6 |
| 6.1 | 0.7 | -3.0 | 1.7 |
| 6.1 | 0.8 | -2.2 | 1.7 |
| 6.1 | 0.9 | -1.6 | 1.7 |
| 6.1 | 1 | -1.1 | 1.6 |
| 6.1 | 2 | 1.0 | 1.2 |
| 6.1 | 4 | -0.8 | 0.5 |
| 6.1 | 6 | 0.3 | 0.2 |
| 8.1 | 0.25 | -9.9 | 0.5 |
| 8.1 | 0.3 | -8.6 | 0.8 |
| 8.1 | 0.35 | -7.6 | 1.0 |
| 8.1 | 0.4 | -6.7 | 1.1 |
| 8.1 | 0.45 | -5.9 | 1.2 |
| 8.1 | 0.5 | -5.2 | 1.4 |
| 8.1 | 0.6 | -4.0 | 1.5 |
| 8.1 | 0.7 | -3.1 | 1.6 |
| 8.1 | 0.8 | -2.4 | 1.6 |
| 8.1 | 0.9 | -1.8 | 1.6 |
| 8.1 | 1 | -1.3 | 1.5 |
| 8.1 | 2 | 0.8 | 1.1 |
| 8.1 | 4 | -0.6 | 0.5 |
| 8.1 | 6 | 0.2 | 0.3 |
| 8.1 | 8 | 0.1 | 0.1 |
| 10.1 | 0.25 | -9.9 | 0.5 |
| 10.1 | 0.3 | -8.7 | 0.7 |
| 10.1 | 0.35 | -7.6 | 0.9 |
| 10.1 | 0.4 | -6.7 | 1.1 |
| 10.1 | 0.45 | -5.9 | 1.2 |
| 10.1 | 0.5 | -5.2 | 1.3 |
| 10.1 | 0.6 | -4.1 | 1.4 |
| 10.1 | 0.7 | -3.2 | 1.5 |
| 10.1 | 0.8 | -2.4 | 1.5 |
| 10.1 | 0.9 | -1.9 | 1.5 |
| 10.1 | 1 | -1.4 | 1.4 |
| 10.1 | 2 | 0.6 | 1.1 |
| 10.1 | 4 | -0.5 | 0.6 |
| 10.1 | 6 | 0.1 | 0.3 |
| 10.1 | 8 | 0.1 | 0.2 |
| 10.1 | 10 | -0.5 | 0.1 |
| 12.1 | 0.25 | -10.0 | 0.4 |
| 12.1 | 0.3 | -8.8 | 0.6 |
| 12.1 | 0.35 | -7.7 | 0.8 |
| 12.1 | 0.4 | -6.8 | 1.0 |
| 12.1 | 0.45 | -6.0 | 1.1 |
| 12.1 | 0.5 | -5.3 | 1.2 |
| 12.1 | 0.6 | -4.2 | 1.3 |
| 12.1 | 0.7 | -3.3 | 1.4 |
| 12.1 | 0.8 | -2.5 | 1.4 |
| 12.1 | 0.9 | -2.0 | 1.4 |
| 12.1 | 1 | -1.5 | 1.4 |
| 12.1 | 2 | 0.5 | 1.0 |
| 12.1 | 4 | -0.4 | 0.6 |
| 12.1 | 6 | 0.1 | 0.3 |
| 12.1 | 8 | 0.0 | 0.2 |
| 12.1 | 10 | -0.3 | 0.1 |
| 14.1 | 0.25 | -10.0 | 0.4 |
| 14.1 | 0.3 | -8.8 | 0.6 |
| 14.1 | 0.35 | -7.8 | 0.8 |
| 14.1 | 0.4 | -6.9 | 0.9 |
| 14.1 | 0.45 | -6.1 | 1.1 |
| 14.1 | 0.5 | -5.4 | 1.2 |
| 14.1 | 0.6 | -4.3 | 1.3 |
| 14.1 | 0.7 | -3.3 | 1.4 |
| 14.1 | 0.8 | -2.6 | 1.4 |
| 14.1 | 0.9 | -2.0 | 1.3 |
| 14.1 | 1 | -1.6 | 1.3 |
| 14.1 | 2 | 0.4 | 1.0 |
| 14.1 | 4 | -0.4 | 0.6 |
| 14.1 | 6 | 0.1 | 0.3 |
| 14.1 | 8 | 0.0 | 0.2 |
| 14.1 | 10 | -0.2 | 0.1 |
| 16.1 | 0.25 | -10.1 | 0.3 |
| 16.1 | 0.3 | -8.9 | 0.5 |
| 16.1 | 0.35 | -7.9 | 0.7 |
| 16.1 | 0.4 | -7.0 | 0.9 |
| 16.1 | 0.45 | -6.2 | 1.0 |
| 16.1 | 0.5 | -5.5 | 1.1 |
| 16.1 | 0.6 | -4.3 | 1.2 |
| 16.1 | 0.7 | -3.4 | 1.3 |
| 16.1 | 0.8 | -2.7 | 1.3 |
| 16.1 | 0.9 | -2.1 | 1.3 |
| 16.1 | 1 | -1.6 | 1.2 |
| 16.1 | 2 | 0.3 | 1.0 |
| 16.1 | 4 | -0.4 | 0.6 |
| 16.1 | 6 | 0.0 | 0.3 |
| 16.1 | 8 | 0.0 | 0.2 |
| 16.1 | 10 | -0.1 | 0.1 |
| 18.1 | 0.25 | -10.1 | 0.3 |
| 18.1 | 0.3 | -8.9 | 0.5 |
| 18.1 | 0.35 | -7.9 | 0.7 |
| 18.1 | 0.4 | -7.0 | 0.8 |
| 18.1 | 0.45 | -6.2 | 0.9 |
| 18.1 | 0.5 | -5.5 | 1.0 |
| 18.1 | 0.6 | -4.4 | 1.2 |
| 18.1 | 0.7 | -3.5 | 1.2 |
| 18.1 | 0.8 | -2.8 | 1.2 |
| 18.1 | 0.9 | -2.2 | 1.2 |
| 18.1 | 1 | -1.7 | 1.2 |
| 18.1 | 2 | 0.3 | 0.9 |
| 18.1 | 4 | -0.4 | 0.6 |
| 18.1 | 6 | 0.0 | 0.4 |
| 18.1 | 8 | 0.0 | 0.2 |
| 18.1 | 10 | -0.1 | 0.1 |
| 20 | 0.25 | -10.1 | 0.3 |
| 20 | 0.3 | -8.9 | 0.5 |
| 20 | 0.35 | -7.9 | 0.7 |
| 20 | 0.4 | -7.0 | 0.8 |
| 20 | 0.45 | -6.2 | 1.0 |
| 20 | 0.5 | -5.5 | 1.1 |
| 20 | 0.6 | -4.4 | 1.1 |
| 20 | 0.7 | -3.5 | 1.2 |
| 20 | 0.8 | -2.8 | 1.2 |
| 20 | 0.9 | -2.2 | 1.2 |
| 20 | 1 | -1.8 | 1.2 |
| 20 | 2 | 0.2 | 0.9 |
| 20 | 4 | -0.4 | 0.6 |
| 20 | 6 | 0.0 | 0.4 |
| 20 | 8 | 0.0 | 0.2 |
| 20 | 10 | -0.1 | 0.1 |

Errors estimates for the tilt-averaged [0, 10] prolate spheroid target strength (dB) calculated by the Kirchhoff-approximation (KA) and Kirchhoff-ray-mode (KRM) scattering models for a range of prolate spheroids with semi-major dimension *ka* and semi-minor dimension *kb*. Errors are obtained by comparison to the tilt-averaged TS calculated from the prolate spheroid modal series model. These data can be interpolated to yield error estimates for any *ka* and *kb* within the region investigated (0.5 < *ka* ≤ 20, 0.25 < *kb* ≤ 10, and *ka* > *kb*).
